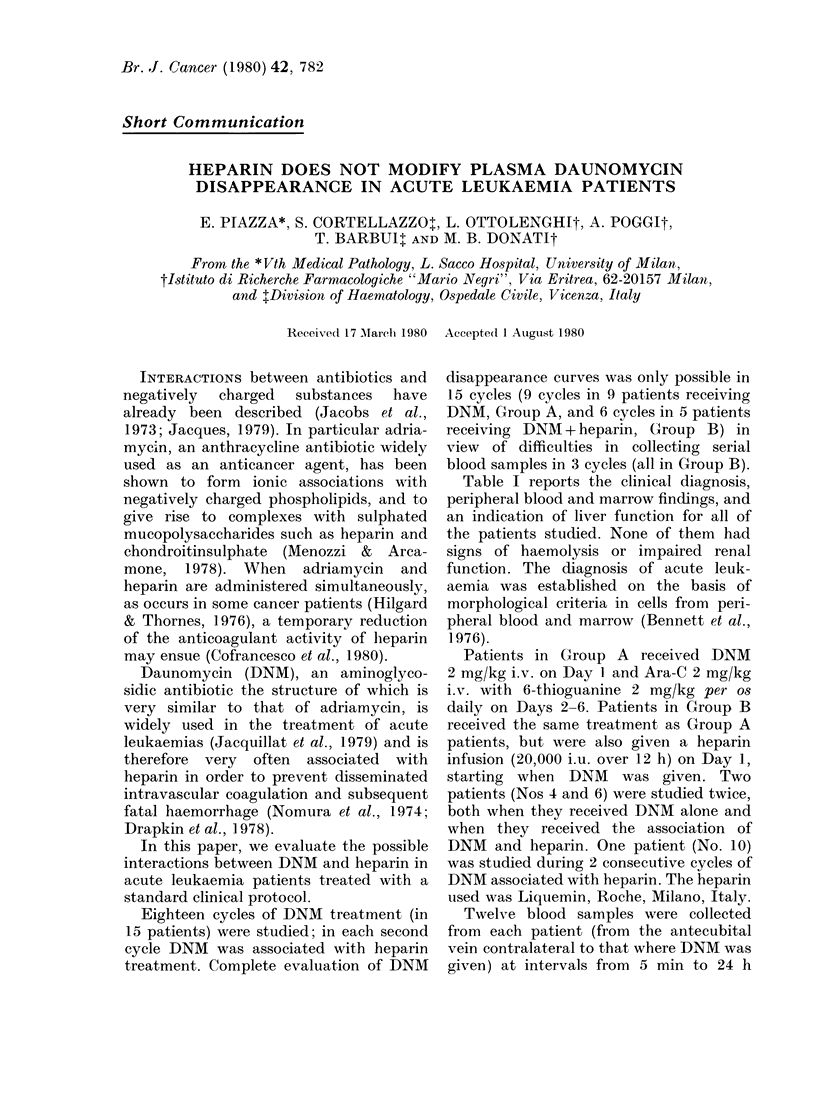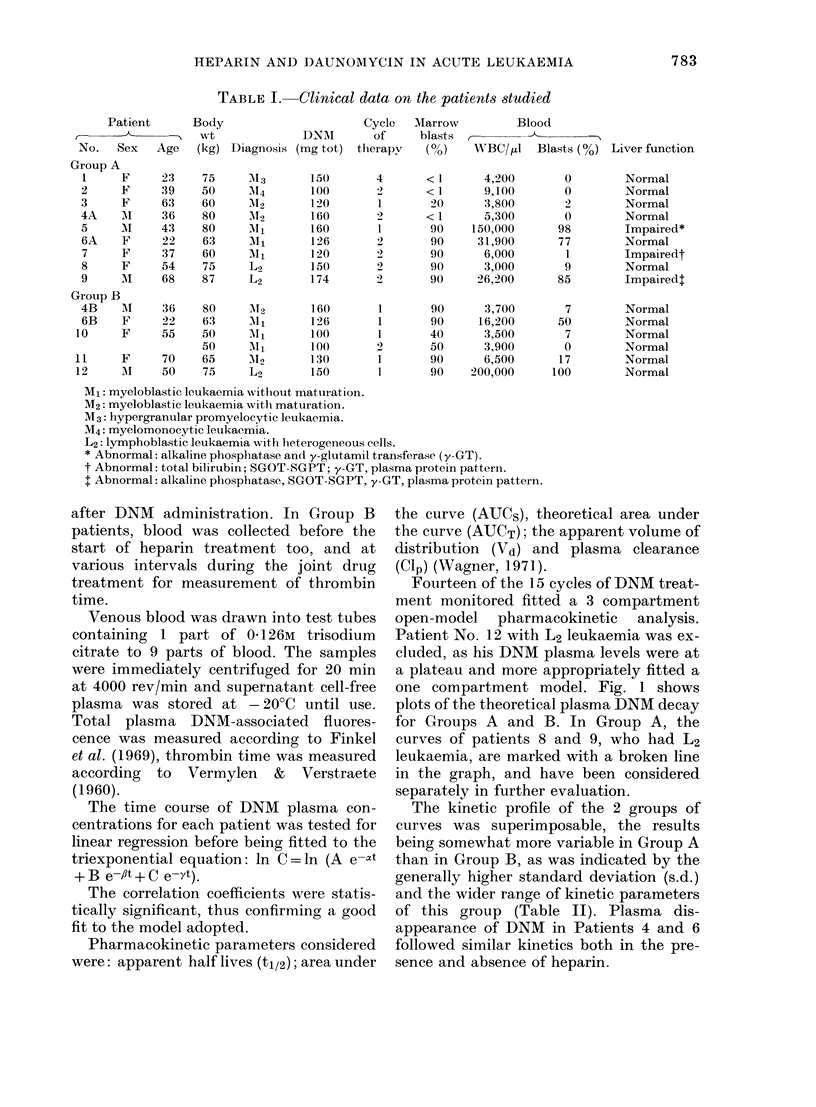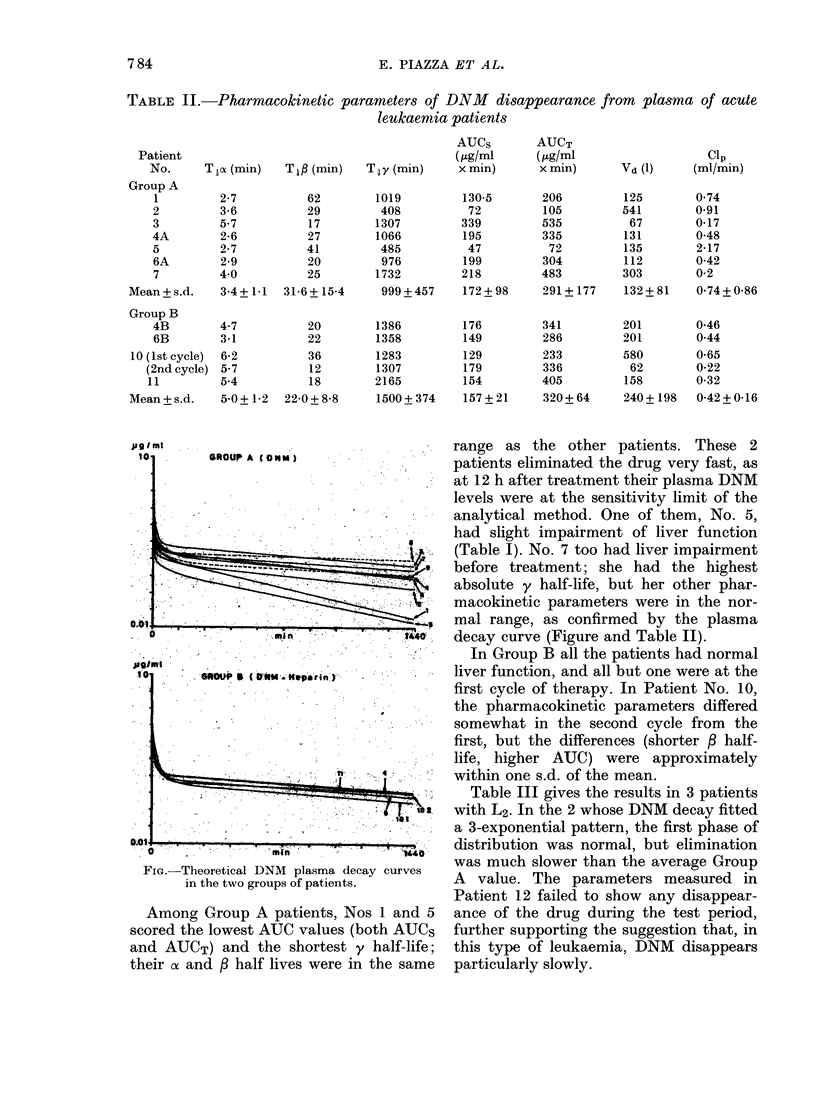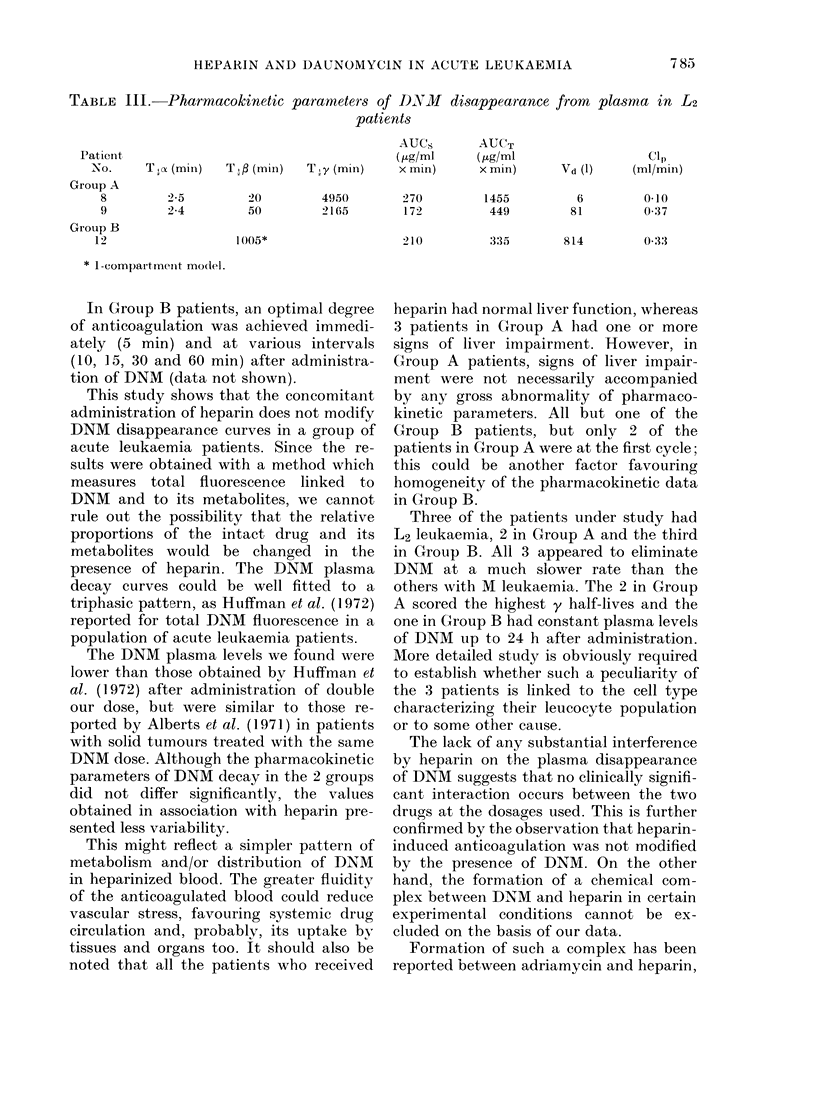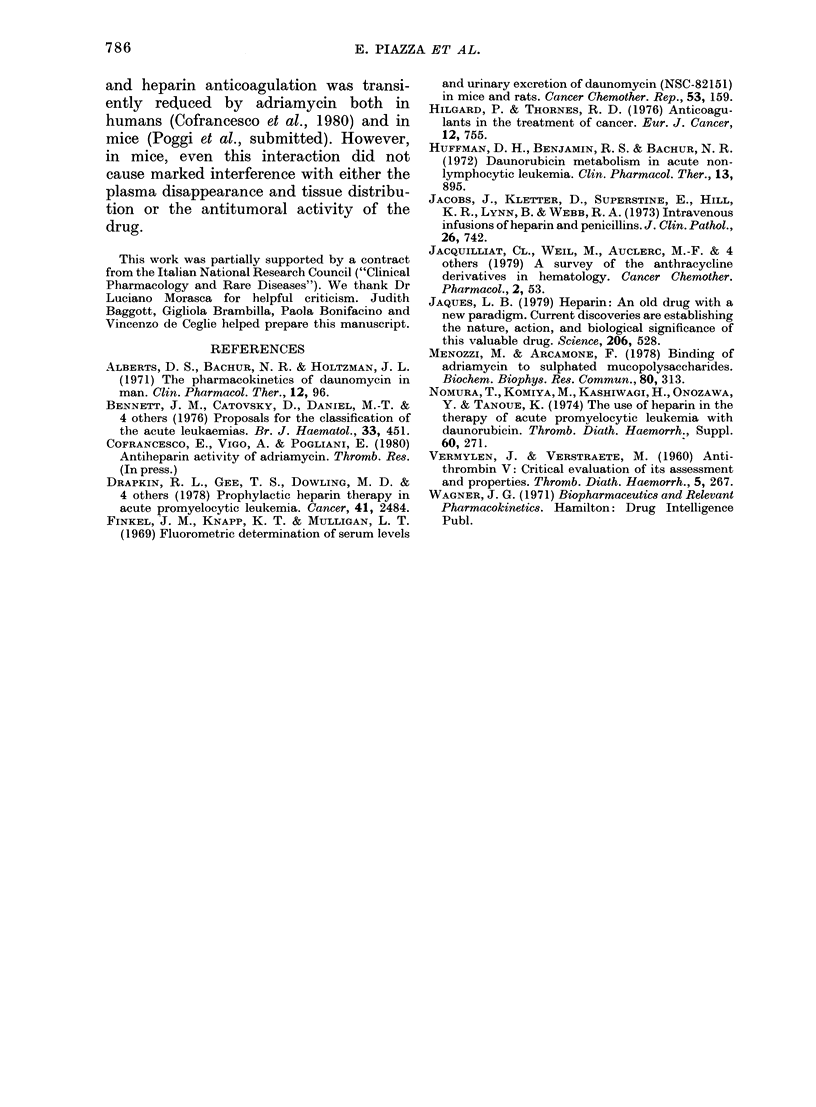# Heparin does not modify plasma daunomycin disappearance in acute leukaemia patients.

**DOI:** 10.1038/bjc.1980.312

**Published:** 1980-11

**Authors:** E. Piazza, S. Cortellazzo, L. Ottolenghi, A. Poggi, T. Barbui, M. B. Donati


*Br. J. Cancer* (1980) **42**, 782

***Short Communication***

### HEPARIN DOES NOT MODIFY PLASMA DAUNOMYCIN DISAPPEARANCE IN ACUTE LEUKAEMIA PATIENTS

E. PIAZZA*, S. CORTELLAZZO‡, L. OTTOLENGHI†, A. POGGI†, T. BARBUI‡ AND M. B. DONATI†

*From the *Vth Medical Pathology, L. Sacco Hospital, University of Milan, †Istituto di Richerche Farmacologiche "Mario Negri", Via Eritrea,* 62-20157 *Milan, and ‡Division of Haematology, Ospedale Civile, Vicenza, Italy*

Received 17 March 1980 Accepted 1 August 1980

INTERACTIONS between antibiotics and negatively charged substances have already been described (Jacobs *et al.*, 1973; Jacques, 1979). In particular adriamycin, an anthracycline antibiotic widely used as an anticancer agent, has been shown to form ionic associations with negatively charged phospholipids, and to give rise to complexes with sulphated mucopolysaccharides such as heparin and chondroitinsulphate (Menozzi & Arcamone, 1978). When adriamycin and heparin are administered simultaneously, as occurs in some cancer patients (Hilgard & Thornes, 1976), a temporary reduction of the anticoagulant activity of heparin may ensue (Cofrancesco *et al.*, 1980).

Daunomycin (DNM), an aminoglycosidic antibiotic the structure of which is very similar to that of adriamycin, is widely used in the treatment of acute leukaemias (Jacquillat *et al.*, 1979) and is therefore very often associated with heparin in order to prevent disseminated intravascular coagulation and subsequent fatal haemorrhage (Nomura *et al.*, 1974; Drapkin *et al.*, 1978).

In this paper, we evaluate the possible interactions between DNM and heparin in acute leukaemia patients treated with a standard clinical protocol.

Eighteen cycles of DNM treatment (in 15 patients) were studied; in each second cycle DNM was associated with heparin treatment. Complete evaluation of DNM disappearance curves was only possible in 15 cycles (9 cycles in 9 patients receiving DNM, Group A, and 6 cycles in 5 patients receiving DNM + heparin, Group B) in view of difficulties in collecting serial blood samples in 3 cycles (all in Group B).

Table I reports the clinical diagnosis, peripheral blood and marrow findings, and an indication of liver function for all of the patients studied. None of them had signs of haemolysis or impaired renal function. The diagnosis of acute leukaemia was established on the basis of morphological criteria in cells from peripheral blood and marrow (Bennett *et al.*, 1976).

Patients in Group A received DNM 2 mg/kg i.v. on Day 1 and Ara-C 2 mg/kg i.v. with 6-thioguanine 2 mg/kg *per os* daily on Days 2–6. Patients in Group B received the same treatment as Group A patients, but were also given a heparin infusion (20,000 i.u. over 12 h) on Day 1, starting when DNM was given. Two patients (Nos 4 and 6) were studied twice, both when they received DNM alone and when they received the association of DNM and heparin. One patient (No. 10) was studied during 2 consecutive cycles of DNM associated with heparin. The heparin used was Liquemin, Roche, Milano, Italy.

Twelve blood samples were collected from each patient (from the antecubital vein contralateral to that where DNM was given) at intervals from 5 min to 24 h

TABLE I.—*Clinical data on the patients studied*

| Patient No. | Sex | Age | Body wt (kg) | Diagnosis | DNM (mg tot) | Cycle of therapy | Marrow blasts (%) | Blood WBC/$\mu$l | Blood Blasts (%) | Liver function |
|---|---|---|---|---|---|---|---|---|---|---|
| Group A | | | | | | | | | | |
| 1 | F | 23 | 75 | $M_3$ | 150 | 4 | < 1 | 4,200 | 0 | Normal |
| 2 | F | 39 | 50 | $M_4$ | 100 | 2 | < 1 | 9,100 | 0 | Normal |
| 3 | F | 63 | 60 | $M_2$ | 120 | 1 | 20 | 3,800 | 2 | Normal |
| 4A | M | 36 | 80 | $M_2$ | 160 | 2 | < 1 | 5,300 | 0 | Normal |
| 5 | M | 43 | 80 | $M_1$ | 160 | 1 | 90 | 150,000 | 98 | Impaired* |
| 6A | F | 22 | 63 | $M_1$ | 126 | 2 | 90 | 31,900 | 77 | Normal |
| 7 | F | 37 | 60 | $M_1$ | 120 | 2 | 90 | 6,000 | 1 | Impaired† |
| 8 | F | 54 | 75 | $L_2$ | 150 | 2 | 90 | 3,000 | 9 | Normal |
| 9 | M | 68 | 87 | $L_2$ | 174 | 2 | 90 | 26,200 | 85 | Impaired‡ |
| Group B | | | | | | | | | | |
| 4B | M | 36 | 80 | $M_2$ | 160 | 1 | 90 | 3,700 | 7 | Normal |
| 6B | F | 22 | 63 | $M_1$ | 126 | 1 | 90 | 16,200 | 50 | Normal |
| 10 | F | 55 | 50 | $M_1$ | 100 | 1 | 40 | 3,500 | 7 | Normal |
| | | | 50 | $M_1$ | 100 | 2 | 50 | 3,900 | 0 | Normal |
| 11 | F | 70 | 65 | $M_2$ | 130 | 1 | 90 | 6,500 | 17 | Normal |
| 12 | M | 50 | 75 | $L_2$ | 150 | 1 | 90 | 200,000 | 100 | Normal |

$M_1$: myeloblastic leukaemia without maturation.
$M_2$: myeloblastic leukaemia with maturation.
$M_3$: hypergranular promyelocytic leukaemia.
$M_4$: myelomonocytic leukaemia.
$L_2$: lymphoblastic leukaemia with heterogeneous cells.
* Abnormal: alkaline phosphatase and $\gamma$-glutamil transferase ($\gamma$-GT).
† Abnormal: total bilirubin; SGOT-SGPT; $\gamma$-GT, plasma protein pattern.
‡ Abnormal: alkaline phosphatase, SGOT-SGPT, $\gamma$-GT, plasma protein pattern.

after DNM administration. In Group B patients, blood was collected before the start of heparin treatment too, and at various intervals during the joint drug treatment for measurement of thrombin time.

Venous blood was drawn into test tubes containing 1 part of 0·126M trisodium citrate to 9 parts of blood. The samples were immediately centrifuged for 20 min at 4000 rev/min and supernatant cell-free plasma was stored at −20°C until use. Total plasma DNM-associated fluorescence was measured according to Finkel *et al.* (1969), thrombin time was measured according to Vermylen & Verstraete (1960).

The time course of DNM plasma concentrations for each patient was tested for linear regression before being fitted to the triexponential equation: $\ln C = \ln (A\ e^{-\alpha t} + B\ e^{-\beta t} + C\ e^{-\gamma t})$.

The correlation coefficients were statistically significant, thus confirming a good fit to the model adopted.

Pharmacokinetic parameters considered were: apparent half lives ($t_{1/2}$); area under the curve ($AUC_S$), theoretical area under the curve ($AUC_T$); the apparent volume of distribution ($V_d$) and plasma clearance ($Cl_p$) (Wagner, 1971).

Fourteen of the 15 cycles of DNM treatment monitored fitted a 3 compartment open-model pharmacokinetic analysis. Patient No. 12 with $L_2$ leukaemia was excluded, as his DNM plasma levels were at a plateau and more appropriately fitted a one compartment model. Fig. 1 shows plots of the theoretical plasma DNM decay for Groups A and B. In Group A, the curves of patients 8 and 9, who had $L_2$ leukaemia, are marked with a broken line in the graph, and have been considered separately in further evaluation.

The kinetic profile of the 2 groups of curves was superimposable, the results being somewhat more variable in Group A than in Group B, as was indicated by the generally higher standard deviation (s.d.) and the wider range of kinetic parameters of this group (Table II). Plasma disappearance of DNM in Patients 4 and 6 followed similar kinetics both in the presence and absence of heparin.

TABLE II.—*Pharmacokinetic parameters of DNM disappearance from plasma of acute leukaemia patients*

| Patient No. | $T_{\frac{1}{2}}\alpha$ (min) | $T_{\frac{1}{2}}\beta$ (min) | $T_{\frac{1}{2}}\gamma$ (min) | $AUC_S$ ($\mu$g/ml × min) | $AUC_T$ ($\mu$g/ml × min) | $V_d$ (l) | $Cl_p$ (ml/min) |
|---|---|---|---|---|---|---|---|
| Group A | | | | | | | |
| 1 | 2·7 | 62 | 1019 | 130·5 | 206 | 125 | 0·74 |
| 2 | 3·6 | 29 | 408 | 72 | 105 | 541 | 0·91 |
| 3 | 5·7 | 17 | 1307 | 339 | 535 | 67 | 0·17 |
| 4A | 2·6 | 27 | 1066 | 195 | 335 | 131 | 0·48 |
| 5 | 2·7 | 41 | 485 | 47 | 72 | 135 | 2·17 |
| 6A | 2·9 | 20 | 976 | 199 | 304 | 112 | 0·42 |
| 7 | 4·0 | 25 | 1732 | 218 | 483 | 303 | 0·2 |
| Mean ± s.d. | 3·4 ± 1·1 | 31·6 ± 15·4 | 999 ± 457 | 172 ± 98 | 291 ± 177 | 132 ± 81 | 0·74 ± 0·86 |
| Group B | | | | | | | |
| 4B | 4·7 | 20 | 1386 | 176 | 341 | 201 | 0·46 |
| 6B | 3·1 | 22 | 1358 | 149 | 286 | 201 | 0·44 |
| 10 (1st cycle) | 6·2 | 36 | 1283 | 129 | 233 | 580 | 0·65 |
| (2nd cycle) | 5·7 | 12 | 1307 | 179 | 336 | 62 | 0·22 |
| 11 | 5·4 | 18 | 2165 | 154 | 405 | 158 | 0·32 |
| Mean ± s.d. | 5·0 ± 1·2 | 22·0 ± 8·8 | 1500 ± 374 | 157 ± 21 | 320 ± 64 | 240 ± 198 | 0·42 ± 0·16 |

FIG.—Theoretical DNM plasma decay curves in the two groups of patients.

Among Group A patients, Nos 1 and 5 scored the lowest AUC values (both $AUC_S$ and $AUC_T$) and the shortest $\gamma$ half-life; their $\alpha$ and $\beta$ half lives were in the same range as the other patients. These 2 patients eliminated the drug very fast, as at 12 h after treatment their plasma DNM levels were at the sensitivity limit of the analytical method. One of them, No. 5, had slight impairment of liver function (Table I). No. 7 too had liver impairment before treatment; she had the highest absolute $\gamma$ half-life, but her other pharmacokinetic parameters were in the normal range, as confirmed by the plasma decay curve (Figure and Table II).

In Group B all the patients had normal liver function, and all but one were at the first cycle of therapy. In Patient No. 10, the pharmacokinetic parameters differed somewhat in the second cycle from the first, but the differences (shorter $\beta$ half-life, higher AUC) were approximately within one s.d. of the mean.

Table III gives the results in 3 patients with $L_2$. In the 2 whose DNM decay fitted a 3-exponential pattern, the first phase of distribution was normal, but elimination was much slower than the average Group A value. The parameters measured in Patient 12 failed to show any disappearance of the drug during the test period, further supporting the suggestion that, in this type of leukaemia, DNM disappears particularly slowly.

TABLE III.—*Pharmacokinetic parameters of DNM disappearance from plasma in $L_2$ patients*

| Patient No. | $T_{\frac{1}{2}}\alpha$ (min) | $T_{\frac{1}{2}}\beta$ (min) | $T_{\frac{1}{2}}\gamma$ (min) | $AUC_S$ (μg/ml × min) | $AUC_T$ (μg/ml × min) | $V_d$ (l) | $Cl_p$ (ml/min) |
|---|---|---|---|---|---|---|---|
| Group A | | | | | | | |
| 8 | 2·5 | 20 | 4950 | 270 | 1455 | 6 | 0·10 |
| 9 | 2·4 | 50 | 2165 | 172 | 449 | 81 | 0·37 |
| Group B | | | | | | | |
| 12 | | 1005* | | 210 | 335 | 814 | 0·33 |

* 1-compartment model.

In Group B patients, an optimal degree of anticoagulation was achieved immediately (5 min) and at various intervals (10, 15, 30 and 60 min) after administration of DNM (data not shown).

This study shows that the concomitant administration of heparin does not modify DNM disappearance curves in a group of acute leukaemia patients. Since the results were obtained with a method which measures total fluorescence linked to DNM and to its metabolites, we cannot rule out the possibility that the relative proportions of the intact drug and its metabolites would be changed in the presence of heparin. The DNM plasma decay curves could be well fitted to a triphasic pattern, as Huffman *et al.* (1972) reported for total DNM fluorescence in a population of acute leukaemia patients.

The DNM plasma levels we found were lower than those obtained by Huffman *et al.* (1972) after administration of double our dose, but were similar to those reported by Alberts *et al.* (1971) in patients with solid tumours treated with the same DNM dose. Although the pharmacokinetic parameters of DNM decay in the 2 groups did not differ significantly, the values obtained in association with heparin presented less variability.

This might reflect a simpler pattern of metabolism and/or distribution of DNM in heparinized blood. The greater fluidity of the anticoagulated blood could reduce vascular stress, favouring systemic drug circulation and, probably, its uptake by tissues and organs too. It should also be noted that all the patients who received heparin had normal liver function, whereas 3 patients in Group A had one or more signs of liver impairment. However, in Group A patients, signs of liver impairment were not necessarily accompanied by any gross abnormality of pharmacokinetic parameters. All but one of the Group B patients, but only 2 of the patients in Group A were at the first cycle; this could be another factor favouring homogeneity of the pharmacokinetic data in Group B.

Three of the patients under study had $L_2$ leukaemia, 2 in Group A and the third in Group B. All 3 appeared to eliminate DNM at a much slower rate than the others with M leukaemia. The 2 in Group A scored the highest $\gamma$ half-lives and the one in Group B had constant plasma levels of DNM up to 24 h after administration. More detailed study is obviously required to establish whether such a peculiarity of the 3 patients is linked to the cell type characterizing their leucocyte population or to some other cause.

The lack of any substantial interference by heparin on the plasma disappearance of DNM suggests that no clinically significant interaction occurs between the two drugs at the dosages used. This is further confirmed by the observation that heparin-induced anticoagulation was not modified by the presence of DNM. On the other hand, the formation of a chemical complex between DNM and heparin in certain experimental conditions cannot be excluded on the basis of our data.

Formation of such a complex has been reported between adriamycin and heparin, and heparin anticoagulation was transiently reduced by adriamycin both in humans (Cofrancesco *et al.*, 1980) and in mice (Poggi *et al.*, submitted). However, in mice, even this interaction did not cause marked interference with either the plasma disappearance and tissue distribution or the antitumoral activity of the drug.

This work was partially supported by a contract from the Italian National Research Council ("Clinical Pharmacology and Rare Diseases"). We thank Dr Luciano Morasca for helpful criticism. Judith Baggott, Gigliola Brambilla, Paola Bonifacino and Vincenzo de Ceglie helped prepare this manuscript.

### REFERENCES

ALBERTS, D. S., BACHUR, N. R. & HOLTZMAN, J. L. (1971) The pharmacokinetics of daunomycin in man. *Clin. Pharmacol. Ther.*, **12**, 96.

BENNETT, J. M., CATOVSKY, D., DANIEL, M.-T. & 4 others (1976) Proposals for the classification of the acute leukaemias. *Br. J. Haematol.*, **33**, 451.

COFRANCESCO, E., VIGO, A. & POGLIANI, E. (1980) Antiheparin activity of adriamycin. *Thromb. Res.* (In press.)

DRAPKIN, R. L., GEE, T. S., DOWLING, M. D. & 4 others (1978) Prophylactic heparin therapy in acute promyelocytic leukemia. *Cancer*, **41**, 2484.

FINKEL, J. M., KNAPP, K. T. & MULLIGAN, L. T. (1969) Fluorometric determination of serum levels and urinary excretion of daunomycin (NSC-82151) in mice and rats. *Cancer Chemother. Rep.*, **53**, 159.

HILGARD, P. & THORNES, R. D. (1976) Anticoagulants in the treatment of cancer. *Eur. J. Cancer*, **12**, 755.

HUFFMAN, D. H., BENJAMIN, R. S. & BACHUR, N. R. (1972) Daunorubicin metabolism in acute nonlymphocytic leukemia. *Clin. Pharmacol. Ther.*, **13**, 895.

JACOBS, J., KLETTER, D., SUPERSTINE, E., HILL, K. R., LYNN, B. & WEBB, R. A. (1973) Intravenous infusions of heparin and penicillins. *J. Clin. Pathol.*, **26**, 742.

JACQUILLIAT, CL., WEIL, M., AUCLERC, M.-F. & 4 others (1979) A survey of the anthracycline derivatives in hematology. *Cancer Chemother. Pharmacol.*, **2**, 53.

JAQUES, L. B. (1979) Heparin: An old drug with a new paradigm. Current discoveries are establishing the nature, action, and biological significance of this valuable drug. *Science*, **206**, 528.

MENOZZI, M. & ARCAMONE, F. (1978) Binding of adriamycin to sulphated mucopolysaccharides. *Biochem. Biophys. Res. Commun.*, **80**, 313.

NOMURA, T., KOMIYA, M., KASHIWAGI, H., ONOZAWA, Y. & TANOUE, K. (1974) The use of heparin in the therapy of acute promyelocytic leukemia with daunorubicin. *Thromb. Diath. Haemorrh.*, Suppl. **60**, 271.

VERMYLEN, J. & VERSTRAETE, M. (1960) Antithrombin V: Critical evaluation of its assessment and properties. *Thromb. Diath. Haemorrh.*, **5**, 267.

WAGNER, J. G. (1971) *Biopharmaceutics and Relevant Pharmacokinetics*. Hamilton: Drug Intelligence Publ.